# Prosocial Norms as a Positive Youth Development Construct: A Conceptual Review

**DOI:** 10.1100/2012/832026

**Published:** 2012-05-01

**Authors:** Andrew M. H. Siu, Daniel T. L. Shek, Ben Law

**Affiliations:** ^1^Department of Rehabilitation Science, The Hong Kong Polytechnic University, Hong Kong; ^2^Department of Applied Social Sciences, The Hong Kong Polytechnic University, Hong Kong; ^3^Department of Social, Work and Social Administration, The University of Hong Kong, Hong Kong

## Abstract

Prosocial norms like reciprocity, social responsibility, altruism, and volunteerism are ethical standards and beliefs that youth development programs often want to promote. This paper reviews evolutionary, social-cognitive, and developmental theories of prosocial development and analyzes how young people learn and adopt prosocial norms. The paper showed that very few current theories explicitly address the issue of how prosocial norms, in form of feelings of moral obligations, may be challenged by a norm of self-interest and social circumstances when prosocial acts are needed. It is necessary to develop theories which put prosocial norms as a central construct, and a new social cognitive theory of norm activation has the potential to help us understand how prosocial norms may be applied. This paper also highlights how little we know about young people perceiving and receiving prosocial norms and how influential of school policies and peer influence on the prosocial development. Lastly, while training of interpersonal competence (e.g., empathy, moral reasoning, etc.) was commonly used in the youth development, their effectiveness was not systematically evaluated. It will also be interesting to examine how computer and information technology or video games may be used in e-learning of prosocial norms.

## 1. Introduction

Social norms are rules and expectations with which a society guides the behavior of its members [1]. Social norms could be very powerful in shaping behavior, as people do not just act in their own interests but also because of conformity to social norms. In general, social norms can be put into two general categories of rules with moral significance (mores) and rules for causal interactions (folkways) which guide our social behavior. As a kind of social norms, prosocial norms are unambiguous, healthy, ethical standards, beliefs, and behavior guidelines that promote prosocial behavior and minimize health hazards [2]. The promotion of prosocial norms is a common objective of positive youth development programs [3]. The prosocial norms that youth development programs often aim to promote include reciprocity, responsibility, volunteerism, and altruism [4]. This paper aims to review the nature, origins, and theories of prosocial norms and how young people learn, evaluate, and adopt prosocial norms. Implications for further research in the youth development will be presented.

Many prevalent prosocial norms are acquired in early life through social learning and identification with role models. These norms often include “reciprocity” which suggests that people should help those who help them and the norm of “social responsibility” which suggests that we should assist people who need help or who depend on us [5]. By being responsible, a person accepts moral and social responsibility and has the ability to make a prosocial decision and a corresponding action that concern issues of justice, rights, and the welfare of others [6]. The concepts of reciprocity and social responsibility can be extended to the citizenship and social contract in some countries—one has the obligation to take care of their own people. However, the boundary of one's social responsibility can be sometimes hard to define, and it is hard to determine how far people should be responsible for their own social groups. Furthermore, there are also norms for “not helping”, as we may learn that it is wiser to “mind your own business” in some social groups, such as in the workplace [7].

Altruism is a selfless concern for the welfare and wellbeing of others who are not related to us in any ways. Some defines it as the antonym of selfishness and egoism. Altruism is closely linked to or embedded in the ethical doctrine of many religions and cultures, which advocate that everybody has a moral obligation to benefit others. Volunteerism is often considered a kind of expression of altruism. Volunteers devote their time to providing services to others without payment. Volunteerism is also regarded as a kind of moral resources and can significantly contribute to the social capital in civil societies. 

The learning, evaluation, and adoption of prosocial norms in children and young people are closely linked to their development of prosocial behaviors such as cooperating, sharing, helping, comforting, donating, volunteering, and taking responsibility [7]. This behavior is often crucial to the cultivation of bonding and quality relationships in social groups, in the maintenance of a harmonious society, and can become a tremendous human resource, such as volunteers for organization of world events (like the Beijing Olympics). 

Among a wide-ranging prosocial behavior, the helping behavior tends to attract the greatest conceptual and empirical interest [8]. There is a growing interest in the conceptualization and scientific study in prosocial development around the world, as prosocial behavior of individuals clearly contributes to the solidarity, economic, and civic development of modern societies. Helping and volunteering behavior does not only benefit others but is often beneficial to the helpers themselves. Helpers experience substantial needs satisfaction and positive influence on their wellbeing [9]. The volunteering experience is widely known to be valuable to youth development. Volunteers can learn and model prosocial norms, understand the world, gain career-related and leadership experience, and strengthen social competence and social relationships [10]. Responsibility behavior is associated with positive relationships with others, high academic achievement, and more positive self-worth in young people [6]. Furthermore, the adoption of prosocial norms and development of prosocial behavior are regarded as incompatible with aggressive or antisocial behavior. In youth development programs, prosocial norms are often promoted alongside behavior guidelines for young people that encourage them to refrain from antisocial behavior like taking drugs, shoplifting, or playing truant from school [3].

## 2. Theories of Prosocial Norms

In the literature, a number of theories have been proposed to explain how children and young people develop and adopt prosocial norms. First, the evolutionary perspective addresses the fundamental question about the origins of prosocial norms and motivation—whether “people are selfish or selfless by nature.” Second, social psychology experiments show that the activation of prosocial norms is greatly influenced by social circumstances like potential costs and rewards and perceived vulnerability of self in providing help to others. Third, theories of developmental psychology postulate that young people with higher levels of moral reasoning and empathy are more likely to be prosocial when compared with those who have lower levels. Fourth, social learning model suggests that the influence stemming from family, peers, and school plays an important part in shaping the adoption of prosocial norms in children and young people. 

First, it appears that people should be selfish under the notion of “survival of the fittest” if we adopt the evolutionary perspective. Being prosocial is a waste of time and energy on the survival of others instead of oneself. Prosocial behavior (like helping) is often against self-interest in the short-term; however, it could have long-term benefits for the survival of the kinship, community, and society. The cultural transmission and internalization of prosocial norms is crucial to the survival of communities and societies. In general, human beings are much more likely to sacrifice themselves for their families, and friends, or groups with which they identified, but than with other groups that they are not related to. Some recent studies have expanded the evolutionary perspective and focused on the role of prosocial emotions, including shame, guilt, empathy, and sympathy in shaping prosocial norms. Prosocial emotions are defined as a genetically grounded, physiologically based system that prepares individuals to obtain rewards from altruistic behavior and expect penalties for selfish behavior in their communities [11]. From the results of some recent experiments, it appears that shame and guilt can be a much more powerful motivator for adhering to prosocial norms (e.g., citizen responsibility to vote) than sympathy, empathy, or pride of doing a moral good [12]. The evolutionary perspective provides a distinct perspective in explaining the origins of motivation for prosocial acts and why human beings are willing to make sacrifice to promote the survival of kinship and community. While there are some initial results on the role of prosocial emotions, there is only partial empirical support for the heritability of prosocial behavior [13]. Time-specific environmental influences tend to contribute a lot more to individual differences in prosocial orientation and behavior. The study of prosocial emotions is an excitingly new development in the evolutionary perspective, but the initial results are pointing to the fact that guilt and shame are far more important than positive emotion of empathy and compassion in prosocial behavior.

Second, from the social psychology literature, we learn that the willingness to provide help (a major form of prosocial behavior) varies according to a number of conditions. The tension reduction models suggest that people help others in pain or distress, in order to relieve their own tension. Cost-reward models also appear to be a plausible explanation of how people decide to act prosocially or not [14]. When we see somebody in need of help, we will often consider the seriousness of the situation, the perceived costs of helping for self, the potential for rewards and commendations, and our own vulnerability in the course of help (especially it involves an emergency) [15]. Social experiments also demonstrate that “bystander effects” can stop the activation of prosocial norms. People are less likely to provide help when there are many “bystanders” who are close to the person who needs help, and a diffusion of responsibility occurs [16]. On the whole, people are also less likely to provide help to strangers if the helping episode is not considered an emergency, if there are many people at the scene, if helping is perceived as costly to the helper, and if we feel that we will make ourselves vulnerable through helping the other person. The social psychology perspective addresses the situational determinants of how people may react differently to others in need (especially strangers) and tells us that prosocial norms may only be applied under “certain” conditions. The “bystander effect” reveals how alienation in a metropolitan life may stop the activation of prosocial norms, and the cost-reward model portraits people as mainly acting in their own interest. These social experiments are revealing, but still do not manage to explain very well why people have to volunteer or make sacrifice for people they do not know.

Third, empirical studies in developmental psychology show that adolescents who adopt more mature and internalized moral reasoning and have higher empathy are more likely to follow and adopt norms of social responsibility and engaged in prosocial behavior [17]. Many studies are based on Kohlberg's developmental stages of prosocial reasoning [18]: (1) hedonistic, self-centered orientation; (2) needs of others orientation; (3) approval orientation and stereotyped orientation; (4) empathic orientation; (5) internalized orientation [18]. In the course of adolescence, young people gradually replace their hedonistic, needs-oriented, and approval-oriented moral reasoning with higher-level prosocial reasoning (e.g., stereotypic, internalized reasoning mode) and perspective-taking skills. At around the same time, the development of perspective taking, role taking, and empathy enables young people to vicariously experience other people's needs and distress [19]. This will lead to a stronger motivation to reduce the distress of others as well as the distress and guilt of oneself for not providing help—the empathy-altruism hypothesis [20]. The developmental approach assumes that the maturity of cognitive abilities will result in an increase of the ability to take perspective of others and to apply internalized moral reasoning and empathy. The approach has accumulated a wealth of evidence that the maturity of these interpersonal competencies is connected to prosocial orientation or behavior. The developmental approach tends to pay less attention to how social cognitive factors and influence may alter the willingness to apply empathy or moral reasoning, especially when one's peers are unsupportive of prosocial norms.

Fourth, prosocial norms can be acquired and taught through social learning, including modeling, social reinforcement, and school socialization [21]. Children and adolescents can learn prosocial norms and behavior from their preferred role models who can demonstrate that how the adoption of prosocial norms and orientation can lead to appreciation, an increase in task-related self-efficacy, and favorable outcome expectancies. In the course of childhood, parents can model emotional expressivity, sympathy, and perspective taking, which can play an important part in the prosocial development among children [22]. Praise, affirmation, encouragement, and social pressure from teachers and peers can provide the incentive and support for adopting prosocial norms. In studies of the youth development and volunteerism, the “foot-in-the-door” socialization technique was found to be very useful in promoting prosocial reasoning [23]. Based on cognitive dissonance theory, the foot-in-the-door technique postulates that there is a strong tendency for us to adjust our attitude in order to make it consistent with what we have done; that is, we justify our choices of prosocial actions after we are required to “put our foot” into it [24]. Parents, schools, or (and) youth centers can require adolescents to fulfill prosocial tasks and responsibilities, which will gradually help to modify their attitudes (become more positive) toward prosocial norms and behavior. 

On the whole, it is apparent that modeling, instructions, and discipline communication of parents may have an impact on prosocial development. Studies of social influence in adolescents often focus on the development of antisocial rather than prosocial norms or behavior. There were even fewer studies on how high school socialization, policies, and discipline may influence the adoption of prosocial norms and values [25]. Social influence encompasses a wide range of sources, including role models but also youth culture, the internet, and the media [26]. Studies of social influence on prosocial norms or orientation can often only examine one of these facets (e.g., parents, school, media, internet, and youth culture) at one time.

All the four theories discussed above are popular theories of development of prosocial motivation, orientation, and behavior, and none of them explicitly regards prosocial norms as a central construct. The norm activation model is a notable social cognitive theory in explaining how prosocial norms play a significant role in prosocial behavior [27, 28]. The model postulates that several variables mediate the relationship between prosocial norms (called personal norms in their model) and prosocial intentions and behavior. “Personal norms” are feelings of a moral obligation to engage in prosocial behavior. Using structural equation modeling, Steg and de Groot successfully fit the variables of problem awareness, ascription of responsibility, and perceived control over the problems as important mediators in the expression of prosocial norms. Problem awareness refers to how far one is aware of negative consequences for others or for other things one values if they do not act prosocially, like there will be more pain and distress for the person in need. The ascription of responsibility is described as feelings of responsibility for the negative consequences of not acting prosocially, such as shame, guilt, and regret. 

These results of the norm activation model implies that the moral obligation to engage in prosocial behavior can be reinforced by an increased awareness of possible emotional and practical consequences of not acting prosocially. However, the possibility if they would express prosocial norms through action depends on their self-efficacy in undertaking the prosocial action effectively and making a difference in resolving the issue. From these empirical results, the norm activation model has identified a few key social cognitive processes on how prosocial norms may be activated when there may be a need for prosocial behavior.

## 3. Prosocial Development in Adolescence

Many prevalent prosocial norms are acquired in early life through social learning and identification with role models. A number of studies support that prosocial orientation and behavior appear in early childhood [29] and peak in late childhood or early adolescence [30]. For instance, studies showed that sharing and generosity gradually increase from mid-childhood to early adolescence [31], while the likelihood of provided emergency intervention behavior has an inverted U-shaped relationship to age. In late childhood, the likelihood to provide emergency help comes to a peak and then decreases again in early adolescence. 

There are plentiful studies on factors contributing to prosocial orientation or behavior from adolescence to early adulthood. Empathy, perspective taking (or role taking), and prosocial moral reasoning are generally identified as key competencies which develop rapidly in adolescence and support the development of a prosocial orientation. In particular, the longitudinal studies by Eisenberg et al. [30] showed that the overall level of prosocial moral reasoning generally increases between 11 and 20 years of age. Hedonistic reasoning (orientation to benefit self) decreases with age, while needs-oriented reasoning (attending to others' needs) increases till late childhood and then remains at a stable level. Direct reciprocal and approval reasoning (conformity with social reinforcement) increases to a peak in mid-adolescence and then remains at a stable level. Forms of higher-level prosocial reasoning (empathic and internalized) emerge in mid- to late adolescence and early adulthood, and there is evidence that the development of moral reasoning is closely linked to the development of prosocial dispositions like sympathy, empathy, and perspective taking [30].

Empathy is another significant ability which is positively connected to prosocial behavior and negatively linked to aggression in early adolescence [32]. A more recent study with Chinese adolescents has showed that empathy increases significantly from 12 to 15 years old [33], which may fuel the increase of prosocial behavior in early adolescence.

Family and school influence is known to play an important role in shaping prosocial norms in childhood [34]. In particular, mothers contribute more strongly to the prosocial development of both sons and daughters. Mothers with an authoritative style, internal attributions for prosocial behavior, and positive responses to prosocial behavior will facilitate the development of prosocial norms in children. However, peer relationships and influence play an increasingly important role in the differentiation of prosocial and antisocial behavior. Teacher influence on prosocial norms and behavior appears to be gradually replaced by peer influence in early to mid-adolescence. Furthermore, peer influence tends to promote delinquent behavior much more than prosocial behavior [35]. 

Studies of peer group dynamics show that adolescents exhibit much more prosocial acts towards “in-group” members than “out-group” members, disregard of how much trust they have on the in-group members [36]. It is also interesting to know that adolescents with higher social anxiety and jealousy in peer relationships tend to exhibit more prosocial acts, so that they can be more easily accepted by peer groups [37]. Adolescents can acquire norms like “experimenting with drugs is a normal adolescent experience” or “adults can't be trusted”. Young people, who have strong bonds to social units like prosocial peers, families, schools, and community, tend to be less involved in problem behaviors. Youth development programs in schools need to impart clear-cut norms and boundaries against problem behaviors and promote young people's commitment to valued relationships in prosocial groups [38]. It is therefore paramount to cover issues of peer influence in youth development programs, such as how to apply one's moral reasoning skills in the face of peer pressure, how to evaluate incentives and sanctions when following group norms, and how to make choices when one's personal values conflict with group norms.

## 4. Gender Issues

Studies around the world consistently show that girls have higher prosocial orientation and more prosocial behaviors than boys throughout adolescence [39, 40]. However, there appears to be no consistent pattern of sex difference in prosocial behavior in the course of adolescence and adulthood [37, 41]. Lots of researchers suggest that gender differences in prosocial behavior can be due to the different body build of males and females, as well as gender role socialization. It is apparent that men and women have widely shared gender role beliefs and “specialize” in different prosocial behaviors. Women are more engaged in acts of caring and support, while men are more engaged in collective-oriented, strength-intensive, and “heroic” actions [42]. This probably reflects a division of labor that develops out of a biosocial interaction based on differences in physical characteristics and social roles of males and females. 

Males and females may also have rather different perceptions of what is meant by “prosocial” [43]. Males may tend to adopt a “justice perspective” that relies on formal moral rules to judge what is prosocial. Females tend to adopt a “care and responsibility perspective,” and prosocial acts should enhance social harmony or reinforce loyalties. Furthermore, competition can be a major barrier to conforming to prosocial norms and behavior for boys, but not for girls [44]. In a competitive situation, boys are more concerned with outcomes of competition than the welfare of others, and they tend to be less proactive in offering help or sharing with others [45]. Instructors of youth development programs need to be sensitive to gender differences and use case examples that take into account the justice perspective of prosocial orientation in boys and the care and responsibility perspective in girls.

## 5. Cultural Issues

A prosocial orientation is highly valued in the traditional Chinese philosophy. Confucian thought encourages people to be kind to others (the practice of *ren*) and seek social harmony [46]. It is regarded as a sign of maturity when a person is able to extend his or her understanding and concern to others. This is expressed in such maxims as “examine others' views by putting yourself into others' position” (*tui ji ji ren* ), “compare people's hearts with your own” (*jiang xin bi ji*), or “do not do to others what you would not wish others to do to you” (*ji suo bu yu, wu shi yu ren*). However, it is significant to note that these concepts are perceived as a kind of “imperfect duty” in Chinese culture, meaning that it is a “lack of virtue” (not good enough) if one does not observe prosocial norms. It is also noteworthy that many of these principles were laid down in feudal times when they were essential for the functioning of collectivistic culture [47]. 

In modern Chinese societies, a prosocial orientation is still highly valued, but it is clear that there are many other possible social, economic, and cultural forces which shape moral standards and prosocial norms [48]. It is even clearer that when individual achievements and wealth are highly valued in modern Chinese societies, pragmatic values can become a lot more popular than prosocial values. Nonetheless, youth development programs can still attempt to make good use of materials from Chinese literature, philosophy, and folklore to help young people realize the duty of a citizen and the virtues put forward in traditional Chinese culture.

## 6. Implications for Research

This paper reviews the theories, prosocial development in the course of adolescence, and the gender and social factors linked to its development. A number of research implications can be drawn from the present discussion. First, most of the existing studies on prosocial behavior and some are on prosocial motivation, but there are relatively few studies on prosocial norms. While it is important to know how far people are engaged in prosocial acts, it is necessary to examine the motivation behind these acts. Researching on prosocial norms will give us some answers on what alters the expression of our prosocial intentions, as there is a wide gap between what one thinks is right and what one actually ends up doing. 

Second, there are only a handful of theories that centered on prosocial norms. Few existing theories really address how people may negotiate between a norm of self-interest and prosocial norms or the feeling of doing what is morally right [49]. Based on social cognitive theory, the norm activation model has attempted to examine how one's feelings of moral obligations may be altered by awareness of consequences for self and others if one does not act prosocially, and the self-efficacy in managing the situation. There are some initial successes in fitting alternative models using the structural equation modeling, and it will be good to continue with this research endeavor and see how this model can be applied under different social circumstances and on different social issues.

Third, reciprocity, social responsibility, altruism, and volunteerism are the most common prosocial norms. People of different age groups or cultural backgrounds may have different interpretations of these norms. We know very little about how and when children and young people learn them. It will be good to conduct some qualitative study using the grounded theory to examine what these norms mean to young people and how far the prosocial norms are compatible with current youth culture.

Fourth, there have been lots of studies on parental influence on prosocial norms and behavior but there have been much less studies on how school and peers may influence or alter the course of prosocial development [25, 50]. In particular, school influence on prosocial norms tends to receive very little attention. It will be interesting to examine school policies, systems of discipline, and commendations that may play a role in shaping prosocial norms and behavior. To obtain a bigger picture of social influence on prosocial norms, some studies can gear towards examining how prosocial norms are transmitted or inhibited through the media, movies and videos, internet, or (and) popular youth culture.

Last but not least, there have been few studies on the effectiveness of specific interventions in youth programs on prosocial norms. Specifically, the training of empathy, perspective taking, prosocial reasoning, and classes on social responsibility has been popular recommendations for enhancing the prosocial development. How can these abilities be taught in schools or youth development activities? It will also be interesting to examine how e-learning may be used in teaching prosocial norms. Reviewing the latest evidence, some researchers suggest that prosocial video games can be effective in shaping prosocial norms and behavior [51]. When young people are becoming more and more engaged with the internet, is it possible to teach prosocial norms through this medium?

## 7. Conclusion

Prosocial norms like reciprocity, social responsibility, altruism, and volunteerism are ethical standards and beliefs that youth development programs often desire to enhance. This paper shows that most of the current theories in prosocial development focus on prosocial behavior rather than on prosocial norms. It is clear from the theories that there are multifaceted influences on the prosocial development, but few theories address the issue of how prosocial norms (in form of feelings of moral obligations) may be deactivated by a norm of self-interest, when prosocial acts appear to be necessary. More theoretical development is needed, and new social cognitive theories of norm activation have the potential to provide some answers to these questions. This paper also highlights that we know very little on how young people perceive and receive prosocial norms (e.g., social responsibility, altruism, etc.) on the school and peer influence on the prosocial development. Lastly, while training of interpersonal competence (e.g., empathy, moral reasoning, etc.) is commonly used in the youth development, their effectiveness is not systematically evaluated. It will also be intriguing to examine how computer and information technology or video games may be used in e-learning of prosocial norms.
